# The General Population’s Perspectives on Implementation of Artificial Intelligence in Radiology in the Western Region of Saudi Arabia

**DOI:** 10.7759/cureus.37391

**Published:** 2023-04-10

**Authors:** Basem H Bahakeem, Sultan F Alobaidi, Amjad S Alzahrani, Roudin Alhasawi, Abdulkarem Alzahrani, Wed Alqahtani, Lujain Alhashmi Alamer, Bassam M Bin Laswad, Nasser Al Shanbari

**Affiliations:** 1 Department of Medical Imaging, College of Medicine, Umm Al-Qura University, Makkah, SAU; 2 Department of Medicine and Surgery, College of Medicine, Umm Al-Qura University, Makkah, SAU

**Keywords:** consensus, artificial intelligence readiness, acceptance, decision making, medical artificial intelligence, diagnosis, community, attitude, view, machine learning

## Abstract

Background

Artificial intelligence (AI) is a broad spectrum of computer-executed operations that mimics the human intellect. It is expected to improve healthcare practice in general and radiology in particular by enhancing image acquisition, image analysis, and processing speed. Despite the rapid development of AI systems, successful application in radiology requires analysis of social factors such as the public’s perspectives toward the technology.

Objectives

The current study aims to investigate the general population’s perspectives on AI implementation in radiology in the Western region of Saudi Arabia.

Methods

A cross-sectional study was conducted from November 2022 and July 2023 utilizing a self-administrative online survey distributed via social media platforms. A convenience sampling technique was used to recruit the study participants. After obtaining Institutional Review Board approval, data were collected from citizens and residents of the western region of Saudi Arabia aged 18 years or older.

Results

A total of 1,024 participants were included in the present study, with the mean age of respondents being 29.6 ± 11.3. Of them, 49.9% (511) were men, and 50.1% (513) were women. The comprehensive mean score of the first four domains among our participants was 3.93 out of 5.00. Higher mean scores suggest being more negative regarding AI in radiology, except for the fifth domain. Respondents had less trust in AI utilization in radiology, as evidenced by their overall distrust and accountability domain mean score of 3.52 out of 5. The majority of respondents agreed that it is essential to understand every step of the diagnostic process, and the mean score for the procedural knowledge domain was 4.34 out of 5. The mean score for the personal interaction domain was 4.31 out of 5, indicating that the participants agreed on the value of direct communication between the patient and the radiologist for discussing test results and asking questions. Our data show that people think AI is more effective than human doctors in making accurate diagnoses and decreasing patient wait times, with an overall mean score of the efficiency domain of 3.56 out of 5. Finally, the fifth domain, “being informed,” had a mean score of 3.91 out of 5.

Conclusion

The application of AI in radiologic assessment and interpretation is generally viewed negatively. Even though people think AI is more efficient and accurate at diagnosing than humans, they still think that computers will never be able to match a specialist doctor’s years of training.

## Introduction

In 1956, John McCarthy, a professor of computer science, was the first to coin the term “artificial intelligence” (AI) [1]. Today, AI is a generic term used to describe the capabilities and functions of machines (computers) that mimic or emulate human intelligence [2]. AI’s technique is deep learning based on a structure of neural networks broadly inspired by the human brain [3]. The field of radiology is a strong candidate for the early integration of AI into the healthcare system since AI has recently shown rapidly improved performance and is moving quickly into the implementation phase in many fields [4]. Furthermore, AI is expected to significantly enhance the quality, value, and breadth of radiology’s contribution to patient care [4]. Although medical imaging has become an essential component in the decision-making process of patient care and despite the availability of technological tools, radiologists nevertheless make mistakes in imaging interpretation that may have serious consequences for the patient [5]. A previous study conducted in the United States showed 1,269 errors among 656 cases, with an average of 251 days from the first misinterpretation to the correct diagnosis [5]. The efficiency, errors, and manual radiologists’ intervention would all be improved by an AI-integrated imaging workflow; therefore, significant efforts and policies have been developed to facilitate the use of AI-related technology in medical imaging [3]. Despite the rapid advancement of AI systems, radiologists must analyze social factors, such as the public’s perspectives on the technology, to successfully apply AI in their field [6]. Thus far, there has been little engagement with patients affected by the application of AI in healthcare, which is alarming because patient concerns about AI may be a significant obstacle to adopting these tools [7]. A recent study in the Netherlands found that 77.8% of 922 participants agreed that a human check was necessary [8]. In addition, a second study of 229 patients in Germany found that 96.2% of patients preferred the physician’s opinion to AI, and physician-supervised AI was deemed to be more acceptable than AI that was not supervised by a physician [9].

To date, no study has measured the general population’s views on the implementation of AI in radiology in Saudi Arabia. To address this issue, we conducted an electronic survey to investigate the general population’s perspectives on AI implementation in radiology in the western region of Saudi Arabia.

## Materials and methods

Study design and participants

This cross-sectional study investigated the general population's perspectives on AI implementation in radiology in the Western region of Saudi Arabia. Convenience sampling was used to recruit the participants. All citizens and residents currently living in the Western region of Saudi Arabia, aged 18 and older, were included in this study. Participants who refused to participate in the study or did not meet the eligibility requirements were excluded.

Ethical considerations and sample size

The study was conducted between November 2022 and July 2023. After obtaining IRB approval from Umm Al-Qura University's Biomedical Ethics Committee (Approval No. HAPO-02-K-012-2022-11-1302), the data were obtained via an online self-administered questionnaire in the Arabic language designed by Google Forms and distributed among the general population. The sample size was calculated using the OpenEpi calculator, and 385 participants were considered the appropriate sample size [10]. The overall sample size was increased to a maximum of 1,000 participants in case of potential data loss and to generalize the study results more efficiently.

Study tool and scoring

The questionnaire form was divided into two different parts: the first part gathered participants' demographic data, including five questions (age, gender, education, specialty or career, and previous radiological imaging), and the second part assessed participants' attitudes toward AI implementation in radiology (composed of 49 attitudinal questions among five key domains: distrust and accountability [15 items], procedural knowledge [18 items], efficiency [5 items], personal interaction [7 items], and being informed [4 items]). A 5-point Likert-type agree-disagree scale was used. Higher scores suggested being more negative regarding AI in radiology except for being informed domain since items' scores in this domain do not reflect a positive or negative attitude regarding AI in radiology. The questionnaire was quoted from a previously published study. An initial pilot study with 30 participants was conducted to assess the validity of the Arabic version of the questionnaire [6]. The data from the pilot study were excluded from the final dataset used in the study.

Statistical analysis

The data were collected, reviewed, and then fed to SPSS version 21 (IBM Corp., Armonk, NY). All statistical methods used were two-tailed with an alpha level of 0.05, which was considered significant if the p-value was less than or equal to 0.05. Regarding participants’ perspectives toward AI and its role in radiology, the overall mean score was obtained for each domain, and the overall attitudinal mean score was calculated based on domains 1-4. Domain 5 was excluded from the calculation since it contains items that do not directly assess the direction of attitude toward AI in radiology. Descriptive analysis was done by prescribing frequency distribution and percentage for study variables, including participants’ socio-demographic data, career specialty, and history of undergoing X-rays. Participants’ perspectives toward AI in radiology were tabulated, and the overall attitudinal mean score for the domains was graphed. Cross-tabulation was used for showing the distribution of participants’ overall attitude scores using mean with SD by their personal data and other factors using ANOVA and independent t-test.

## Results

A total of 1,024 eligible participants completed the study questionnaire. Participants ages ranged from 18 to 70 years, with a mean age of 29.6 ± 11.3. Of the participants, 511 (49.9%) were male. Regarding educational level, 629 (61.4%) were university graduates, 311 (30.4%) had a secondary level of education, and 64 (6.3%) had a postgraduate degree. A total of 314 (30.7%) worked in the medical field, 108 (10.5%) were IT/computer science engineers, and 602 (58.8%) were in other specialties. A total of 765 (74.7%) reported that they had had an X-ray before (Table 1).

**Table 1:** Socio-demographic data of the study participants in the Western region of Saudi Arabia (n = 1024).

Socio-demographic data	No.	%
Age in years		
18-20	133	13.0%
21-25	384	37.5%
26-30	161	15.7%
31-40	143	14.0%
> 40	203	19.8%
Mean ± SD	29.6 ± 11.3	
Gender		
Male	511	49.9%
Female	513	50.1%
Educational level		
Below secondary	20	2.0%
Secondary/diploma	311	30.4%
University	629	61.4%
Post-graduate	64	6.3%
Study/Work specialty		
Medical field	314	30.7%
IT/computer science	108	10.5%
Others	602	58.8%
Have you had an X-ray before?		
Yes	765	74.7%
No	259	25.3%

Table 2 shows that 70% of participants agreed that AI could only be implemented to check human judgment; 67.2% agreed that even if computers are better at evaluating scans, they still prefer a doctor; 63.1% felt that humans have a better overview than computers on what happens in their bodies; 63.1% found it worrisome that a computer does not take feelings into account; and 61.7% would never blindly trust a computer. On the other hand, 56% of participants thought AI might prevent errors, and 51.5% thought AI would replace doctors in the future.

**Table 2:** Participants' attitude towards AI's distrust and accountability domain (n = 1024). AI: Artificial intelligence.

Distrust and accountability	Strongly disagree		Disagree		Neutral		Agree		Strongly agree	
	No.	%	No.	%	No.	%	No.	%	No.	%
A computer can never compete against the experience of a specialized doctor	35	3.4%	121	11.8%	297	29.0%	353	34.5%	218	21.3%
Through human experience, a radiologist can detect more than the computer	22	2.1%	123	12.0%	257	25.1%	391	38.2%	231	22.6%
Humans have a better overview than computers on what happens in my body	27	2.6%	131	12.8%	220	21.5%	384	37.5%	262	25.6%
It worries me when computers analyze scans without interference of humans	51	5.0%	170	16.6%	246	24.0%	363	35.4%	194	18.9%
I wonder how it is possible that a computer can give me the results of a scan	86	8.4%	234	22.9%	255	24.9%	289	28.2%	160	15.6%
Artificial intelligence makes doctors lazy	141	13.8%	226	22.1%	218	21.3%	250	24.4%	189	18.5%
I think radiology is not ready for implementing artificial intelligence in evaluating scans	66	6.4%	221	21.6%	335	32.7%	257	25.1%	145	14.2%
I think replacement of doctors by artificial intelligence will happen in the far future	92	9.0%	185	18.1%	220	21.5%	362	35.4%	165	16.1%
I would never blindly trust a computer	43	4.2%	133	13.0%	216	21.1%	372	36.3%	260	25.4%
Artificial intelligence can only be implemented to check human judgment	31	3.0%	93	9.1%	183	17.9%	421	41.1%	296	28.9%
I find it worrisome that a computer does not take feelings into account	59	5.8%	118	11.5%	201	19.6%	353	34.5%	293	28.6%
It is unclear to me how computers will be used in evaluating scans	28	2.7%	142	13.9%	264	25.8%	398	38.9%	192	18.8%
Even if computers are better in evaluating scans, I still prefer a doctor	22	2.1%	93	9.1%	221	21.6%	399	39.0%	289	28.2%
When artificial intelligence is used, my personal data may fall into the wrong hands	56	5.5%	162	15.8%	259	25.3%	344	33.6%	203	19.8%
Artificial intelligence may prevent errors	32	3.1%	103	10.1%	316	30.9%	393	38.4%	180	17.6%

A total of 91% of participants found it essential to have a good understanding of the results of a scan. Consistently, 89.6% found it essential to be able to ask questions personally about the results of a scan, 87.6% found it essential that a scan provide as much information about their body as possible, 87.3% found it necessary to talk with someone about the results of a scan, and 86.9% found it important to ask questions about the reliability of the results. About 78.9% of participants found it important to be well-informed about how a scan is made (Table 3).

**Table 3:** Participants' attitude towards AI's procedural knowledge domain (n = 1024). AI: Artificial intelligence.

Procedural knowledge	Strongly disagree		Disagree		Neutral		Agree		Strongly agree	
	No.	%	No.	%	No.	%	No.	%	No.	%
I find it important to have a good understanding of the results of a scan	4	0.4%	8	0.8%	80	7.8%	396	38.7%	536	52.3%
I find it important to be able to ask questions personally about the results of a scan	8	0.8%	15	1.5%	84	8.2%	356	34.8%	561	54.8%
I find it important to talk with someone about the results of a scan	5	0.5%	22	2.1%	103	10.1%	328	32.0%	566	55.3%
I find it important that a scan provides as much information about my body as possible	10	1.0%	25	2.4%	92	9.0%	342	33.4%	555	54.2%
I find it important to get the results of a scan as fast as possible	17	1.7%	23	2.2%	120	11.7%	365	35.6%	499	48.7%
I find it important to ask questions on the reliability of the results	10	1.0%	17	1.7%	107	10.4%	350	34.2%	540	52.7%
I find it important to be well informed about how a scan is made	7	0.7%	39	3.8%	170	16.6%	344	33.6%	464	45.3%
I find it important to read how radiologists work before I get a scan	9	0.9%	23	2.2%	118	11.5%	356	34.8%	518	50.6%

An exact 88.2% of participants said that when discussing the results of a scan, humans are indispensable; 87.5% found it necessary to ask questions when getting a result; 86.2% needed to be treated as a person, not as a number; and 85% thought that humans and AI could complement each other. A total of 76.3% of participants thought that getting the results should involve personal contact (Table 4).

**Table 4:** Participants' attitude towards AI's personal interaction domain (n = 1024). AI: Artificial intelligence.

Personal interaction	Strongly disagree		Disagree		Neutral		Agree		Strongly agree	
	No.	%	No.	%	No.	%	No.	%	No.	%
When discussing the results of a scan, humans are indispensable	9	0.9%	26	2.5%	86	8.4%	304	29.7%	599	58.5%
Getting the results involves personal contact	12	1.2%	34	3.3%	197	19.2%	323	31.5%	458	44.7%
As a patient, I want to be treated as a person, not as a number	11	1.1%	19	1.9%	111	10.8%	290	28.3%	593	57.9%
When a computer gives the result, I would miss the explanation	16	1.6%	39	3.8%	146	14.3%	344	33.6%	479	46.8%
I find it important to ask questions when getting the result	13	1.3%	25	2.4%	90	8.8%	323	31.5%	573	56.0%
Even when computers are used to evaluate scans, humans always remain responsible	13	1.3%	29	2.8%	124	12.1%	347	33.9%	511	49.9%
Humans and artificial intelligence can complement each other	24	2.3%	28	2.7%	102	10.0%	322	31.4%	548	53.5%

A total of 75.4% of participants agreed that evaluating scans with AI would reduce healthcare waiting times; 55.9% thought that the sooner they got the results, even when from a computer, the more they would be at ease. An exact 52.9% of participants reported that fewer doctors and radiologists are required because of AI, and 49.5% thought that humans make more errors than computers (Table 5).

**Table 5:** Participants' attitude towards AI's efficiency domain (n = 1024) AI: Artificial intelligence.

Efficiency	Strongly disagree		Disagree		Neutral		Agree		Strongly agree	
	No.	%	No.	%	No.	%	No.	%	No.	%
As far as I am concerned, artificial intelligence can replace doctors in evaluating scans	67	6.5%	189	18.5%	338	33.0%	267	26.1%	163	15.9%
The sooner I get the results, even when this is from a computer, the more I am at ease	34	3.3%	117	11.4%	301	29.4%	347	33.9%	225	22.0%
Because of the use of artificial intelligence, fewer doctors and radiologists are required	70	6.8%	146	14.3%	266	26.0%	356	34.8%	186	18.2%
Evaluating scans with artificial intelligence will reduce healthcare waiting times	17	1.7%	51	5.0%	184	18.0%	465	45.4%	307	30.0%
In my opinion, humans make more errors than computers	46	4.5%	81	7.9%	390	38.1%	311	30.4%	196	19.1%

A total of 75.1% of participants thought that when a computer can predict whether they will get a disease in the future, they would want to know that information no matter what, and 72.2% reported that if it does not matter in costs, a computer should always make a full body scan instead of looking at specific body parts, while 62.3% thought that a computer should only look at body parts that their doctor selected. Of the respondents, 70.4% thought that if a computer gave the results, they would not feel emotionally supported (Table 6).

**Table 6:** Participants' attitude towards being informed using AI technology (n = 1024). AI: Artificial intelligence.

Being informed	Strongly disagree		Disagree		Neutral		Agree		Strongly agree	
	No.	%	No.	%	No.	%	No.	%	No.	%
If it does not matter in costs, a computer should always make a full body scan instead of looking at specific body parts	27	2.6%	74	7.2%	184	18.0%	359	35.1%	380	37.1%
If a computer would give the results, I would not feel emotional support	27	2.6%	59	5.8%	217	21.2%	402	39.3%	319	31.2%
A computer should only look at body parts that were selected by my doctor	29	2.8%	103	10.1%	254	24.8%	393	38.4%	245	23.9%
When a computer can predict that I will get a disease in the future, I want to know that no matter what	32	3.1%	51	5.0%	172	16.8%	354	34.6%	415	40.5%

**Figure 1:**
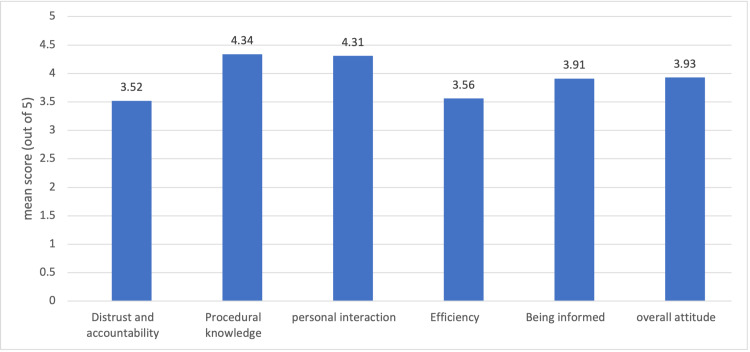
The overall mean score for each domain and the overall attitudinal means score (n = 1024). The overall attitudinal score was calculated based on factors 1-4; factor 5 was excluded from the calculation since it contains items that do not directly assess the direction of attitude toward AI in radiology.

Figure 1 describes the means of the five domains and the overall mean of the first four domains. The highest score was for procedural knowledge (4.34 out of 5), followed by personal interaction (4.31 out of 5), while the lowest score was for AI efficiency (3.56 out of 5). The comprehensive mean score of the first four domains among our participants was 3.93 out of 5.00 (Figure 1).

The highest score was detected among participants aged 31-40 years (4.00 ± 0.34) and lowest among others aged 21-25 years (3.86 ± 0.45) with recorded statistical significance (p = 0.001). Also, participants who had previously undergone an X-ray showed significantly higher mean attitude scores than others who did not (3.95 ± 0.43 vs. 3.87 ± 0.41, respectively; p = 0.007; Table 7).

**Table 7:** Factors affecting the population's overall perspectives toward artificial intelligence implementation in radiology. P: One Way ANOVA; $: Independent t-test; * P < 0.05 (significant).

Socio-demographic data	Overall attitude level		P-value
	Mean	SD	
Age in years			0.001*
18-20	3.88	0.38	
21-25	3.87	0.45	
26-30	3.90	0.46	
31-40	4.00	0.34	
> 40	3.93	0.43	
Gender			0.099^$^
Male	3.91	0.45	
Female	3.95	0.40	
Educational level			0.491
Below secondary	4.04	0.53	
Secondary/Diploma	3.90	0.41	
University	3.94	0.41	
Post-graduate	3.94	0.58	
Student/Work specialty			0.310
Medical field	3.90	0.40	
IT/Computer science	3.93	0.47	
Others	3.95	0.43	
Have you had an X-ray before?			0.007*^$^
Yes	3.95	0.43	
No	3.87	0.41	

## Discussion

The use of AI in medical imaging could have various benefits, including improved efficiency and patient satisfaction with the diagnostic process, shorter wait times, and greater confidence in the diagnosis [11]. AI is expected to soon become more critical in radiology [3]. In the literature, patient attitudes toward AI in healthcare have been discussed; however, little attention has been paid to its application in radiology [6,12,13]. Our goal was to investigate the general population's expectations regarding the application of AI in radiology, which is essential for developing AI systems. In accordance with what we expected based on the literature, our data suggest that people generally have negative attitudes toward the application of AI in radiology (overall score of 3.93) [6,7,14]. However, many studies have reported the opposite, in which people were generally positive about utilizing AI to evaluate their radiology reports. A potential reason for this is the difference in educational levels and technical affinity among the population [15,16]. With an average score of 3.52 for the first domain (distrust and accountability), our findings indicate that participants favor human judgment over that of AI systems and that people are somewhat against the idea of AI replacing radiologists' diagnostic decisions. These findings are consistent with what was found by Ongena YP et al. study, which reported a roughly similar score for this domain (3.28) [6]. This is not surprising, considering the multiple results of decreased trust in AI for medical practice and the need for physicians to control AI [14,16]. Regarding the second domain (procedural knowledge), respondents agreed with several studies that it is crucial to have a comprehensive understanding of the entire diagnostic process, including how and what data are obtained and processed, scoring an average of 4.34 [6,15]. The third domain (personal interaction) received an average score of 4.31, almost identical to that of Ongena YP et al., suggesting that participants agreed on the importance of personal interaction between the patient and the radiologist for discussing examination results and asking questions [6]. For the fourth domain (efficiency), our data suggest that people believe AI to be more effective than human doctors in making accurate diagnoses and shortening patient wait times (average score of 3.56). This goes against the findings of Ongena YP et al., who showed that patients are on the fence about whether AI will enhance diagnostic workflow, giving an average score of 2.89 [6]. 

Scores on several items within the fifth domain (being informed) varied. People, for instance, prefer AI systems to examine the entire body rather than specific body parts (average score of 3.97), which is consistent with the findings of Ongena YP et al.'s study [6]. To a greater extent, participants in our sample would like to receive information from AI systems about potential future diseases (average score of 4). In contrast, respondents indicated that if computers provided them with results, they would feel a lack of emotional support (mean score: 3.91), which is consistent with the findings of several studies. This makes sense, given that effective patient-doctor communication and empathy are more important to patients than medical outcomes [6,15,17]. 

Limitations 

By using a new tool, our study contributes to a better understanding of public attitudes toward AI in radiology. However, there are still a few limitations. First, electronic surveys were distributed via social media, which, in addition to affecting the credibility of the participants' responses, raises the likelihood of sampling bias. Second, despite the large research sample with an equal male-to-female ratio, the study only reflects a single area in Saudi Arabia, making it difficult to generalize the findings across the country.

## Conclusions

The present study generally reported a negative attitude toward the application of AI in radiologic assessment and interpretation. Although our results reported that people believe AI to be more effective and accurate in diagnosing than humans, they still believe that computers can never compete against the experience of a specialized doctor. They favor human judgment over the AI system. Furthermore, the participants believe personal interaction between the patient and the radiologist is essential. Our findings also revealed the necessity of enhancing AI systems' clarity and providing better and simpler explanations to raise people's confidence and acceptance of AI utilization.
